# DNA Methylation and Intra-Clonal Heterogeneity: The Chronic Myeloid Leukemia Model

**DOI:** 10.3390/cancers13143587

**Published:** 2021-07-17

**Authors:** Benjamin Lebecque, Céline Bourgne, Véronique Vidal, Marc G. Berger

**Affiliations:** 1 Hématologie Biologique, CHU Clermont-Ferrand, Hôpital Estaing, 1 Place Lucie et Raymond Aubrac, CEDEX 1, 63003 Clermont-Ferrand, France; blebecque@chu-clermontferrand.fr (B.L.); cbourgne@chu-clermontferrand.fr (C.B.); 2 Equipe d’Accueil 7453 CHELTER, Université Clermont Auvergne, CHU Clermont-Ferrand, Hôpital Estaing, 1 place Lucie et Raymond Aubrac, CEDEX 1, 63003 Clermont-Ferrand, France; 3 Helixio, 63360 Saint-Beauzire, France; vvidal@helixio.com

**Keywords:** chronic myeloid leukemia, DNA methylation, clonal heterogeneity

## Abstract

**Simple Summary:**

In Chronic Myeloid Leukemia (CML), intra-clonal heterogeneity is a major factor in the response to tyrosine kinase inhibitors and in leukemia stem cell persistence. This intra-clonal heterogeneity could be partially explained by epigenetic abnormalities. This review focuses on DNA methylation abnormalities in CML and its potential implications for the development of new biomarkers of the treatment response and new therapy opportunities. DNA methylation abnormalities are considered an important event in the CML progression phase. Moreover, in recent years, DNA methylation abnormalities have also been characterized at CML diagnosis (in the chronic phase), with specific alterations in the immature cells of the tumor clone. Lastly, the review discusses the importance of these finding for understanding the disease emergence, for developing new therapeutic strategies, and for a personalized management of CML.

**Abstract:**

Chronic Myeloid Leukemia (CML) is a model to investigate the impact of tumor intra-clonal heterogeneity in personalized medicine. Indeed, tyrosine kinase inhibitors (TKIs) target the BCR-ABL fusion protein, which is considered the major CML driver. TKI use has highlighted the existence of intra-clonal heterogeneity, as indicated by the persistence of a minority subclone for several years despite the presence of the target fusion protein in all cells. Epigenetic modifications could partly explain this heterogeneity. This review summarizes the results of DNA methylation studies in CML. Next-generation sequencing technologies allowed for moving from single-gene to genome-wide analyses showing that methylation abnormalities are much more widespread in CML cells. These data showed that global hypomethylation is associated with hypermethylation of specific sites already at diagnosis in the early phase of CML. The BCR-ABL-independence of some methylation profile alterations and the recent demonstration of the initial intra-clonal DNA methylation heterogeneity suggests that some DNA methylation alterations may be biomarkers of TKI sensitivity/resistance and of disease progression risk. These results also open perspectives for understanding the epigenetic/genetic background of CML predisposition and for developing new therapeutic strategies.

## 1. Introduction

Chronic myeloid leukemia (CML) is a unique model of leukemogenesis. Indeed, this disease of middle aged adults (median age at disease onset: 56 years) [1] originates from a single cytogenetic abnormality considered to be the driver of hematopoietic stem cell (HSC) transformation: the translocation t(9;22)(q34;q11) that results in the formation of the Philadelphia (Ph1) chromosome [2]. The resulting chimeric BCR-ABL transcript produces the BCR-ABL fusion protein with constitutive tyrosine kinase activity that leads to uncontrolled granulocytic lineage proliferation, adhesion defects, and resistance to apoptosis. CML is classified in three phases: (i) chronic phase (CP) the natural course of which is the transformation into (ii) the blast phase (Blast Crisis, BC) that resemble acute leukemia and has a poor prognosis [3], and (iii) an intermediate phase, known as the accelerated phase (AP) that may precede the BC. The management of patients is based on the use of tyrosine kinase inhibitors (TKIs), the first targeted therapy in oncology [4], that has revolutionized CML prognosis.

The evaluation of the therapeutic response with a 20-year follow-up and the progress made in residual disease monitoring with the quantification of the chimeric BCR-ABL transcript have allowed standardizing CML management [5]. However, only a minority of patients (≃15%) can be considered in durable remission. Moreover, half of the patients with undetectable residual disease relapse after treatment discontinuation [6,7,8], demonstrating the persistence of TKI-insensitive leukemic stem cells (LSCs) [9,10]. These relapses also are the proof of intra-clonal heterogeneity. Indeed, although all malignant cells harbor the BCR-ABL fusion protein, a small subpopulation resists TKI treatment and persists in vivo. The immunophenotype of this subpopulation is comparable to that of normal hematopoietic stem cells and although it is a minority within the initial clone, its presence demonstrates the existence of intra-clonal heterogeneity. The cells that are more resistant to TKIs seem to be quiescent, again highlighting the variability of cell behaviors within the same clone [11]. BCR-ABL-related resistance mechanisms, such as point mutations, explain only a minority of the cases of primary resistance to TKIs [5], but are representative examples of intra-clonal heterogeneity because cells harboring a BCR-ABL mutation are mostly selected by targeted therapy [12]. Other BCR-ABL-independent mechanisms have been suggested, for example related to the microenvironment [13,14]. Similar to many other cancer types, epigenetic abnormalities also could be involved, notably DNA methylation [15,16].

This review focuses on methylation abnormalities in the CML clone, and their role in intra-clonal heterogeneity and disease progression.

## 2. DNA Methylation Functions in Hematopoiesis

### 2.1. DNA Methylation 

DNA methylation is one of the most described epigenetic mechanism, and it plays an essential role in gene expression regulation and chromatin organization [17]. In this process, a methyl group is added to the fifth carbon of cytosines in CpG sequences to form 5-methylcytosine (5mC). In mammals, DNA methylation is predominantly found at cytosines in symmetrical CpG dinucleotide base pairs. Indeed, 70–80% of them are methylated, although they represent only 3–8% of all cytosines. CpG dinucleotides are enriched in genomic areas called CpG islands (CGIs) that are frequently present at gene promoters and are, therefore, poorly methylated. CGI methylation is often associated with transcription repression and long-term silencing [17].

**Figure 1:**
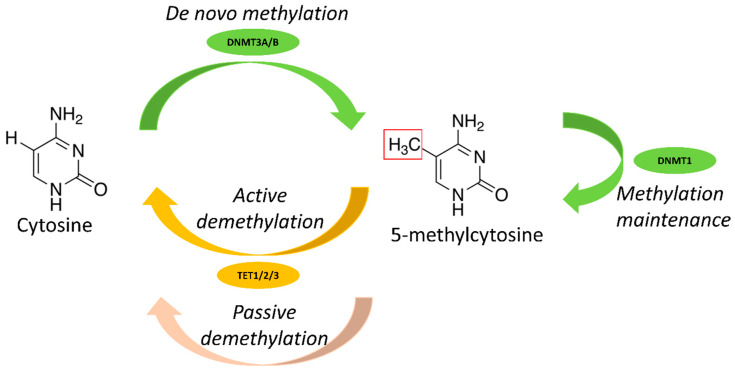
Main enzymes involved in DNA methylation.

DNA methylation is controlled by different enzymes (Figure 1). DNA methyltransferases (DNMTs) [18] catalyze the addition of the methyl group and include three main enzymes: DNMT1, DNMT3A and DNMT3B. DNMT1 is involved in DNA methylation maintenance during DNA replication, whereas DNMT3A and DNMT3B are implicated in de novo methylation. Other enzymes, such as ten-eleven translocation (TET), activation-induced cytidine deaminase (AID) and thymine DNA glycosylase (TDG), play a role in demethylation [19,20,21,22]. DNA methylation is involved in various biological phenomena, for instance the epigenetic regulatory mechanisms of genomic imprinting, X-chromosome inactivation and cell differentiation [23,24], and plays a major role also in hematopoiesis.

### 2.2. DNA Methylation in Hematopoiesis

**Figure 2:**
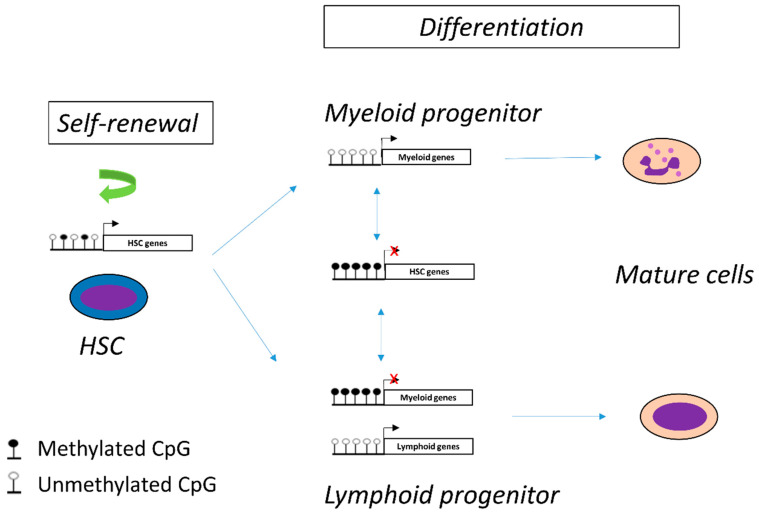
DNA methylation controls hematopoietic cell differentiation and self-renewal of hematopoietic stem cells (HSC).

Hematopoiesis is a dynamic process that proceeds in a hierarchical manner. Multipotent HSCs give rise to oligopotent progenitors that then differentiate into uni-potent progenitors committed towards a specific lineage. This model is finely regulated and controlled. DNA methylation plays an essential role in cell plasticity, lineage commitment and cell differentiation. Indeed, DNA methylation contributes to the maintenance of HSC stemness and participates in cell differentiation [25] (Figure 2). Thus, the methylation profile differs according to the differentiation stage and the cell type. For instance, myeloid progenitors are characterized by lower global DNA methylation than lymphoid cells where DNA methylation silences the myeloid differentiation program [26,27].

The finding that some hemopathies are accompanied by a maturation block while others can maintain cell differentiation suggests that epigenetic mechanisms, particularly alterations in DNA methylation, may be involved in the development of the leukemic phenotype. Consequently, as the CML clone is derived from the leukemic transformation of one HSC [28] and that myeloid differentiation is maintained during the disease, it can be hypothesized that epigenetic alterations, particularly DNA methylation, are involved in leukemogenesis and intra-clonal heterogeneity.

## 3. DNA Methylation Abnormalities in CML

In cancer, DNA methylation abnormalities can be associated with aberrant gene expression. For example, the genome-wide DNA hypomethylation observed in the cancer cell genome has been associated with genomic instability, whereas DNA hypermethylation of CGIs at specific promoters leads to aberrant gene repression [29]. These methylation abnormalities are found at particular genomic locations called cancer-specific differentially methylated regions [30]. Although more limited than in solid tumors, significant DNA methylation changes are also observed in CML.

### 3.1. DNA Methylation Abnormalities of BC-CML Cells

#### 3.1.1. Methylation Abnormalities Are Associated with CML Progression

The mechanisms of transformation from CP to BC-CML are still poorly understood, particularly due to the high genetic heterogeneity and complexity of blast phase cells. Several genetic mechanisms of transformation have been suggested on the basis of the results obtained by comparing primary CML cells in chronic phase and in accelerated/blast phase: (1) increased BCR-ABL1 expression associated with genetic instability [31]; (2) appearance of mutations in the BCR-ABL1 kinase domain, responsible for resistance to TKIs [32,33,34]; (3) appearance of additional cytogenetic abnormalities in 60%–80% of cases [35]; (4) presence of gene mutations, notably in *ASXL1* (tumor suppressor), *RUNX1* (HSC self-renewal), and *IKZF1* (myeloid cell differentiation) [32,33,34]; (5) presence of copy number variations [32,34]; and (6) presence of other fusion genes (26% of patients in the study by Branford et al.) [32].

The first studies on DNA methylation in CML were performed in blast phase cells, known for their genetic instability. The first analyses on a limited number of genes [36,37,38,39,40,41,42], reviewed in [43], suggested the existence of methylation abnormalities in CML. A tendency to DNA hypermethylation was observed in BC compared with CP-CML primary cells. However, this hypermethylation was rarely correlated with a change in the target gene expression level [42]. Technological advances allowed more extensive DNA methylation analyses in parallel with transcriptomic analyses [34,44]. By analyzing 17 CP, 4 AP, 9 BC and 5 control (healthy donor) samples (mononuclear cells from peripheral blood or bone marrow) using the Reduced Representation Bisulfite Sequencing (RRBS) technique, Heller et al. [44] identified approximately 6500 differentially methylated CpG sites in the BC samples compared with controls. They reported that DNA methylation abnormalities were discrete in the early phase of CP and increased in the BC (around 0.3% of abnormally methylated CpG sites analyzed in CP, 1% in AP, and 2% in BC) (see Section 3.2). By RNA-sequencing, they confirmed the link between DNA methylation and downregulation in 22.5% of genes. More recently, Ko et al. [34] performed a methylation analysis (HM450K arrays) and RNA-seq analysis of 7 healthy donors (CD34+ cells from bone marrow), 28 CP (CD34+ cells from peripheral blood and bone marrow) and 30 BC samples (*n* = 18 acute myeloid leukemia and *n* = 12 acute lymphoblastic leukemia; CD34+ cells from peripheral blood and bone marrow). They confirmed that BC transformation is mainly characterized by DNA hypermethylation events (>80%), often at promoters. This can be explained by the fact that these abnormalities could involve areas already methylated in normal and/or CP-CML cells, corresponding to genes that are normally not or only slightly expressed. More indirect regulatory mechanisms, such as the use of an alternative promoter or the presence of a permissive histone mark (such as trimethylation of lysine 4 on histone 3, H3K4me3), could be involved [45].

The mechanisms involved in the progression to BC could affect DNA methylation via, for example, polycomb repressive complexes (PRCs). For instance, PRC-2 and enhancer of zeste homolog 2 (EZH2) might induce the hypermethylation phenotype [34]. However, the link between BCR-ABL1 and PRCs is poorly understood.

#### 3.1.2. Differences and Similarities with Ph1-Negative Acute Myeloid Leukemia (AML)

Many methylation abnormalities have also been detected in Ph1-negative AML. In these hemopathies, different factors may influence the DNA methylation profile. First, the genetic driver abnormalities present in Ph1-negative AML [46,47,48], such as recurrent cytogenetic abnormalities (AML1-ETO, CBFb-MYH11 or PML-RARA) and *MLL* gene rearrangements, are associated with specific DNA methylation profiles [46]. However, inter-individual variability exists within subgroups. This is probably the result of several factors, including age and the presence of additional mutations [49] that do not appear to influence DNA methylation in BC-CML [34]. Second, unlike BC-CML where the lymphoid and myeloid blast phase cells have similar methylation profile [34], Ph1-negative AML is influenced by the cell origin of the clone and the differentiation stage at which the clone stalls [50]. Finally, mutations in DNA methylation enzymes (DNMT3A, TET2, IDH1 and IDH2) have been found in ~44% of AML [48], and only in 16% of AP/BC-CML [51]. These mutations, particularly in DNMT3A and IDH1/2, are associated with specific methylation signatures [48,52,53], and are sometimes identified as a driver of disease development, particularly in AML with normal karyotype [54]. For example, DNA hypermethylation patterns are associated with IDH1 and IDH2 mutations, and hypomethylation with DNMT3A alterations [46,48,55]. DNA methylation can also be altered by mutations in genes of the cohesin complex involved in chromatin organization [54]. The consequences of these DNA methylation abnormalities are variable and multiple: repression of tumor suppressor gene expression, re-expression of a gene that was initially switched off, promotion of the appearance of gene (e.g., *TP53*, *DNMT3A*) mutations, via C-T transitions, necessary for disease initiation or progression [29].

Thus, the genetic mechanisms involved in CML development and blast transformation probably involve epigenetic mechanisms that need to be elucidated due to CML transformation into acute leukemia.

#### 3.1.3. Epigenetics, CML and AML Predisposition

Exposure to toxic substances, such as benzene, can promote the development of AML and CML [56,57], suggesting common leukemogenesis mechanisms. 

Furthermore, in BC-CML, besides the Ph1 chromosome, other chromosomal abnormalities, called additional karyotypic abnormalities, have been observed (e.g., monosomy 7, missing chromosome Y, trisomy of chromosome 8, trisomy of chromosome 21, extra Ph chromosome) [35,58]. These abnormalities are also associated with AML or have poor prognosis [59], but they concern a minority of patients and exceptionally the CP. 

In AML, various mutations in genes involved in the development of myeloid malignancies and constituting predisposition factors (e.g., *CEBPA*, *GATA2*, *RUNX1*, *ANKRD26*, *ETV6*, *ASXL1*) have been identified, particularly from the study of hereditary diseases, familial forms, and karyotypic abnormalities [60]. Conversely in CML, the natural course of which is transformation into acute leukemia, only some of these mutations are detected (e.g., *RUNX1* or *ASXL1*), and they are present only in about half of patients with BC-CML, and in a minority of patients with CP-CML [31,32,33,59,61]. Interestingly, although gene mutations can be present already in the pre-leukemic clone, therapeutic resistance to TKI correlates more with mutations occurring during the follow-up than with their initial presence [62]. The occurrence of these chromosomal/genetic perturbations and/or BCR-ABL point mutations during the progressive phases of the disease might reflect the well-known genetic instability in CML, probably favored by DNA methylation abnormalities [37,38,43,63].

Genetic abnormalities that affect telomere dynamics are predisposed to AML development [60,64].

More recently, mutations characteristic of age-related clonal hematopoiesis of indeterminate potential (CHIP) have been described in healthy subjects [65]. These mutations are found in 10% of >65-year-old subjects, but only in 1% of <50-year-old subjects [66]. These recurrent mutations mainly concern genes involved in epigenetic mechanism regulation (*DNMT3A, TET2, ASXL1, BCOR, IDH1, IDH2*), but also tumor suppressors (*TP53*), genes involved in signaling pathways (*JAK2, CBL*), and spliceosome genes (*SF3B1*) [67,68,69,70]. In healthy subjects, mutations in genes encoding DNA methylation regulators (*DNMT3A, TET2*) are associated with DNA methylation abnormalities [65] that influence the dynamics of hematopoietic differentiation [71]. These mutations are also found in patients with myelodysplastic syndromes or AML. Studies in large samples showed that CHIP is a pre-leukemic state that predisposes to AML development [68,69,72,73,74]. It is also associated with higher risk of mortality, and particularly of cardiovascular diseases [74,75].

CHIP is found in only about 15% of patients (Table 1) with CP-CML at diagnosis, and it is mainly related to *ASXL1, DNMT3A* or *TET2* gene mutations. Two studies suggested that some of these mutations may be present before the diagnosis because they are detected in the Ph-negative clones after TKI treatment [62,76]. They may also be more frequent at diagnosis in patients who progress to BC (50%–60%) [32]. This work suggests that in some patients, CHIP favors progression to AML, but the link between CHIP and CML is not clear.

### 3.2. DNA Methylation in CP-CML and Intra-Clonal Heterogeneity

The first studies on DNA methylation abnormalities at diagnosis of CP-CML focused on a limited number of genes, for instance the promoter Pa of *ABL1* [38,77,78] the hypermethylation of which is now considered one of the characteristics of the DNA methylation pattern of the CML clone. These studies found that the promoters of several genes, such as *PU-1* [79] and *HOXA4* [63], were abnormally methylated at CP diagnosis. Overall, DNA methylation abnormalities were more frequent in the progressive phases of the disease, and methylation changes were proposed as markers of CML progression [34,36,37,38,39,40,41,42,43,44,79,80,81,82] and of therapeutic resistance [38,43,63,83]. Then, the use of more comprehensive methylation profiling approaches revealed more extensive changes, but again, mainly in the progressive phases of the disease [34,44]. Indeed, changes in the CP appear to be minimal (approximately 0.3% of all CGIs) compared with control mononuclear cells, and their relationship with the initial therapeutic response is unclear [44].

**Table 1:** Mutation in genes encoding epigenetic regulators at CP-CML diagnosis (relative to the total number of patients under study).

Genes Investigated	TET1	TET2	TET3	DNMT3A	DNMT1 DNMT3B	ASXL1	Epigenetic Regulator	DNA Methylation
4 genes [84]	/	1/91	/	/	/	8/91	9/91	1/91
25 genes [76]	/	1/15	/	2/15	/	1/15	4/15	3/15
71 genes [85]	0/124	1/124	/	4/124	0/124	9/124	37/124	5/124
92 genes [62]	0/100	6/100	/	2/100	0/100	9/100	19/100	8/100
Whole exome [86]	0/24	1/24	1/24	0/24	0/24	3/24	6/24	2/24
Whole exome [87]	0/40	1/40	0/40	1/40	0/40	6/40	10/40	2/40
Whole exome [32]	0/46	0/46	0/46	0/46	0/46	9/46	11/46	1/46
Total	0/334	11/440	1/110	9/349	0/334	45/440	96/440	22/440
%	0%	2.50%	0.90%	2.60%	0%	10.20%	21.80%	5%

On the basis of the resistance to TKIs of the immature subpopulation of the initial BCR-ABL1-positive CML clone and the maintenance of myeloid differentiation and therefore of cellular heterogeneity, our group hypothesized an initial intra-clonal epigenetic heterogeneity, particularly concerning DNA methylation, an epigenetic marker that can be passed from one cell generation to the next [88]. We used a global methylome analysis approach (HM450k arrays) to analyze immature CD34+ CD15- cells and mature CD34- CD15+ cells of the CML clone at diagnosis, and also the equivalent cell subpopulations from healthy controls [77]. The analysis strategy included a step to remove DNA methylation changes related to myeloid differentiation. With this strategy we showed, for the first time, that the CP-CML clone at diagnosis has a characteristic DNA methylation profile with an overall tendency to hypomethylation (33 CML-specific abnormally methylated hotspots), and also that the immature CD34+ CD15- subpopulation has a specific profile with a more pronounced hypomethylation. The two cell subpopulations show only 65% of similarity. The list of target genes includes genes with already known DNA methylation abnormalities, such as hypermethylation of *ABL1,* and genes that are overexpressed in BC-CML, such as *PRAME, WT1* and *GAS2* [89,90,91]. The existence of an altered DNA methylation profile already at CP-CML diagnosis has been confirmed [92], including in the CD34+ cell subpopulation [34].

Interestingly, among the genes undergoing DNA methylation changes in the CP-CML CD34+ CD15- cell subpopulation, we noticed a significant number of alternatively spliced genes. A recent study demonstrated the existence of abnormal splicing already in the CP of the disease [92].

## 4. Pathophysiology of DNA Methylation Changes

The mechanisms leading to DNA methylation abnormalities are still poorly understood. Although DNA methylation changes are important in the advanced stages of CML [34,44] mutations in DNA methylation regulators are rarely observed [32,33,34,93]. 

The similarity of the methylomes and transcriptomes of the myeloid and lymphoid blast phases is in favor of a minimal impact of the cell orientation on DNA methylation alterations in the acute phase [34], and suggests the existence of crucial epigenetic perturbation steps upstream of the clonal expansion phase that may be related to BCR-ABL1 expression. Indeed, one of the main hypotheses is the reprogramming of the DNA methylation signature by the BCR-ABL fusion protein [94]. 

Vicente-Dueñas et al. [95] demonstrated that expression of the BCR-ABL p210 transcript in mice induces global DNA hypomethylation and DNMT1 overexpression, which can be reduced by the TKI imatinib. In a recent study using a cellular reprogramming strategy to erase the DNA methylation pattern of BCR-ABL+ cell lines, Amabile et al. [94] showed that BCR-ABL1 activation induces an aberrant DNA methylation phenotype that is reversed upon BCR-ABL1 repression. Furthermore, 5-azacytidine, a hypomethylating agent used to treat some hemopathies, can reduce the oncogenic potential of BCR-ABL+ cells in a murine model, as observed with imatinib. These results suggest that the BCR-ABL1 fusion protein can induce a change in DNA methylation, and that DNA methylation abnormalities are necessary for the maintenance of the leukemic phenotype and the oncogenic potential of malignant cells. However, this study used BCR-ABL+ cells from patients with BC-CML and not CP-CML, and did not distinguish between BCR-ABL-dependent and -independent abnormalities. The disappearance of most of the DNA methylation abnormalities identified using a global whole-genome bisulfite sequencing approach in patients with a satisfactory molecular response after TKI treatment suggests a major inductive role of BCR-ABL [92]. However, in patients with optimal therapeutic response, it is difficult to detect possible abnormalities persisting in the small CML cell population surviving in vivo.

On the other hand, several observations suggest that the BCR-ABL fusion protein may not be the only inducer of DNA methylation alterations. First, the initial intra-clonal heterogeneity of the CML clone and the existence of a specific profile of the immature CD34+ subpopulation [34,77] indicate that although BCR-ABL is expressed in all the cells of the clone, the DNA methylation alteration profile is different between immature and mature cells. This suggests the existence of BCR-ABL-independent mechanisms. Furthermore, a possible amplification of the chimeric *BCR-ABL1* gene in CD34+ cells, as demonstrated in TKI-resistant cell lines [96,97], cannot explain a change in the methylation targets of BCR-ABL. Therefore, it is likely that the “immature” status of the cells also plays a role in DNA methylation changes. Second, as these studies did not use the new sequencing techniques that allow for a more comprehensive analysis of methylation abnormalities, they might have missed some alterations. Third, the effect of epigenetically targeted drugs, alone or in combination with TKIs, suggests the existence of therapeutic resistance mechanisms independent of BCR-ABL (see Section 5). 

The most recent studies support a link between PRCs and DNA methylation changes. PRCs primarily target histones and modify their marks, and are essential for HSC and LSC stemness. Unlike other hemopathies, in CML, E2H2 (PRC2 complex) is rarely mutated, but is overexpressed in CP cells [98]. Its exact role remains unclear. As PRC2, which catalyzes trimethylation of lysine 27 on histone H3 (H3k27me3), is recruited to unmethylated CGIs, it is possible that the overall DNA hypermethylation of the CML clone alters the PRC2 target profile. Interestingly, Ko et al. [34] found evidence of a strong relationship between PRCs and methylation changes in the blast phase. At this stage, the overall change is mostly towards DNA hypermethylation, particularly at gene promoters. The DNA sequence of methylated genes is enriched in PRC targets. Using integrative and chromatin immunoprecipitation-sequencing approaches, this group compared the methylation profiles of BC and CP cells. They found stronger binding of EZH2 and BMI1 (another PRC protein) in the CP than BC samples, and many hypermethylated promoters in BC cells. An enrichment in H3k27me3 (repressive mark) and a decrease in H3k4me3 (activating mark) at hypermethylated sites correspond to the canonical PRC effect. Moreover, an enrichment in genes with bivalent chromatin (i.e., with both repressing and activating epigenetic marks) was noted, particularly in BC cells [77]. It seems that EZH2 plays a more important role than BMI1 in the DNA methylation-dependent repression of genes in BC-CML, particularly those involved in differentiation. Thus, PRCs might play a significant role in the methylation profile changes observed in BC cells.

However, the mechanisms of the early DNA methylation alterations in CML cells during leukemogenesis and then in the CP (i.e., global DNA hypomethylation and hypermethylation of specific promoters) remain unknown. 

In summary, besides BCR-ABL-dependent mechanisms, other mechanisms are implicated in the DNA methylation alterations observed in the CML clone and they might be involved in the observed intra-clonal epigenetic heterogeneity (Table 2), and in the mechanisms of resistance of immature cells to targeted therapy. More studies are needed to better understand the pathophysiology of these epigenetic anomalies.

## 5. Perspectives

### 5.1. DNA Methylation, Biological Age, and CML Predisposition

**Figure 3:**
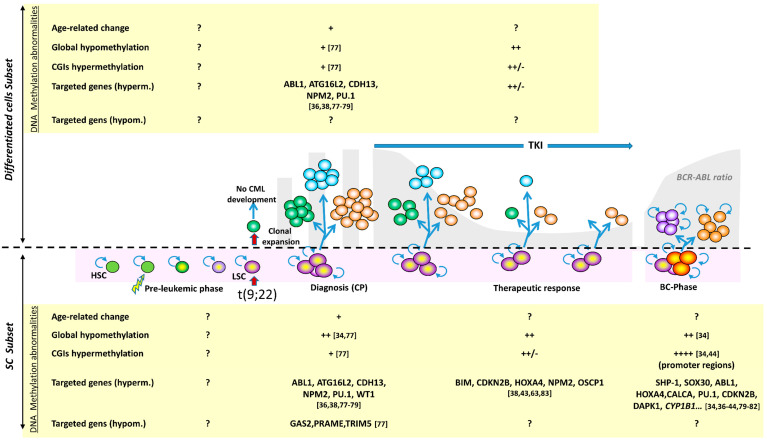
Major methylation alterations in the mature and immature cell compartments during CML course. The longitudinal history of CML is shown with all possible phases: pre-leukemic phase, leukemic transformation of a hematopoietic stem cell (HSC), clonal amplification and diagnosis in the chronic phase, TKI treatment, and blast transformation (now a rare event). A distinction is made between immature cells (SC subset) and mature cells (differentiated cell subset). The most remarkable methylation alterations are indicated for both compartments; when these changes concern the whole clone, without distinction between subsets, the information is indicated for the differentiated cell compartment. For the blast phase, information is provided in the context of immature cells, taking into account the acquisition of stem cell-like characteristics by the progenitors (symbolized by the self-renewal symbol). Only few predisposing factors are known (exposure ionizing radiation, benzene); the set of initial oncogenic factors is symbolized by the lightning arrow. The pre-leukemic phase spreads over several years, is very poorly known, and no information on methylation alterations is available. At diagnosis, the DNA methylation profile is abnormal, with global hypomethylation but CGI hypermethylation in CD34+ cells, and a more balanced distribution of hyper- and hypo- methylated genes. DNA hypermethylation of some genes is consistently observed, some of which represent a typical clonal abnormality, such as *ABL1* hypermethylation. During treatment, the DNA methylation changes related to therapeutic resistance remain poorly studied and concern few genes. In the blast phase, the DNA methylation profile changes abruptly. While globally maintaining the CP abnormalities, hypermethylation accompanies disease progression. The number of the bibliographic references appears in square brackets.

The presence of DNA methylation abnormalities at CP-CML diagnosis raises questions about their potential role in the disease emergence and development (Figure 3).

In subjects exposed to ionizing radiation [99,100], CML will develop 5 to 10 years after irradiation. The genetic and epigenetic events involved in CML emergence during these long years are unknown. This long interval between irradiation and CML could have many reasons, for instance: (i) the slow HSC division rate in vivo, because CML emergence requires the appearance of the translocation (9;22) in one HSC [101]; (ii) the immune system efficiency; (iii) the patient’s sex and age. Specifically, CML incidence is higher in men and increases with age [100]. As epigenetic regulatory mechanisms are involved in cell stemness, immune system and cell ageing, they might also have a significant role in the early stages of leukemogenesis.

As mentioned above, CML is a particularly attractive model to study the relationship between epigenetics and leukemogenesis outside CHIP. One of the major current research axes is the close relationship between DNA methylation, and chronological age, and age-associated pathologies [102,103]. This should provide a more powerful tool for assessing cell ageing than transcriptomic, proteomic or telomere length-based approaches. The first methylation clocks were described by S. Horvath [104] (a multi-tissue profile) and Hannum et al. [105] (blood cell profile). Based on these studies, several algorithms have been developed, some of which take into account factors that influence the biological age [106,107,108].

**Figure 4:**
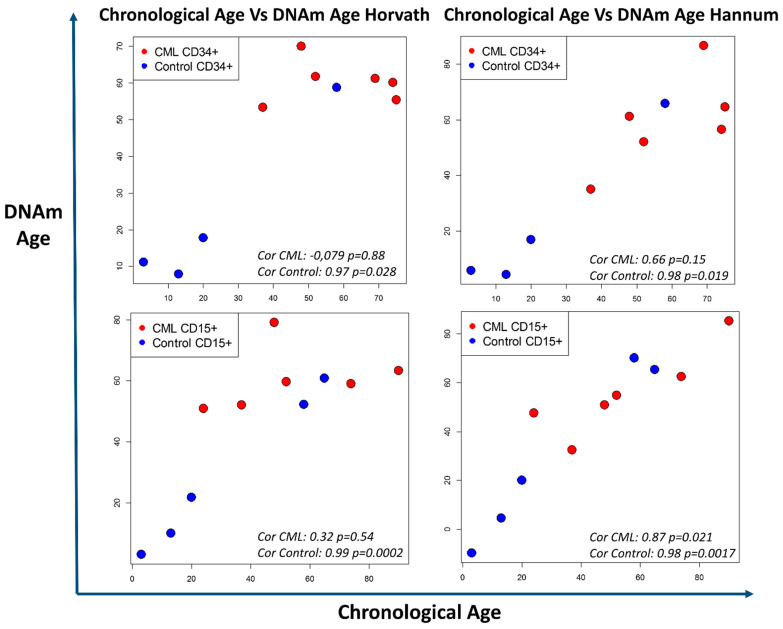
Discrepancy between chronological age and biological age evaluated with two DNA methylation clocks. Methylation data (HM450K chip) from Maupetit-Mehouas et al. [77] were analyzed with the DNAm age calculator based on the algorithms described by Horvath [104] and Hannum [105]. The method and R function is described in [104]. Results are presented in graphical form with chronological age (x-axis) versus the DNA methylation (DNAm) age (y-axis) for CD34+CD15- and CD34-CD15+ CP-CML cells at diagnosis (red dots) and control cells (blue dots) from healthy donors. Overall, chronological age and biological age were comparable in control cells. Conversely, biological age was altered in CP-CML samples, particularly in CD34+CD15- cells, showing accelerated cell aging in the youngest patients and a slower cell aging in older patients. Statistical test: Pearson correlation test.

Consequently, the question of the involvement of biological age (aging) in CML emergence and development appears relevant. Indeed, a disturbance of the biological age has been reported in many cancers [109], but no data are available for CML. Using algorithms to calculate the DNA methylation age, we analyzed again the HM450K microarray data of CP-CML cells and of cells from healthy donors [77] that contain the sites identified by Horvath et al. and Hannum et al.. Overall, we found a correlation between biological and chronological age in control cells. Conversely, we found differences between chronological and biological age in CD34+ CD15- and CD34- CD15+ CP-CML cells (Figure 4; four CP-CML samples paired, two impaired). Aging was accelerated in cells from the youngest patients, but not in cells from the oldest patients. As a result, all patients’ samples showed a similar biological age that roughly corresponded to the median age of disease onset. These results suggest the existence of age-related DNA methylation abnormalities common to all patients, regardless of age. This could be linked to the emergence and amplification of the CML clone and possibly to an individual predisposition. There are discrete differences between CD34+ CD15- and CD34- CD15+ cells that may reflect intra-clonal heterogeneity, but no data has been reported on methylation clock differences within the hemopoietic cell hierarchy or within the same malignant clone. These interesting preliminary results raise the question of the impact of altered cell aging, but they need to be confirmed in a larger patient population and if possible by including also pediatric samples despite the rarity of CML in children (around 10 new cases per year in France).

### 5.2. DNA Methylation as a Therapeutic Target

DNA methylation changes might influence the characteristics of the CML clone by altering the expression of some genes, albeit to a limited extent [34,77,110], or indirectly by affecting genetic instability and promoting the appearance of gene mutations [111]. In addition, DNA methylation changes are increased in patients showing imatinib resistance and during disease progression [36,38,63,80]. Therefore, DNA methylation changes might represent interesting therapeutic targets. DNMT inhibitors and particularly hypomethylating agents, such as azacytidine and decitabine, have a biological impact (viability, proliferation) on K562 and KCL22 myelogenous cells and primary CD34+ BC-CML cells [34,44,112], unpublished personal data]. Moreover, Amabile et al. [94] showed that 5-azacytidine significantly decreases the malignant potential of BCR-ABL+ cells in a mouse model of CML. This indicates that DNA methylation alterations participate in the leukemic phenotype induced by the BCR-ABL transcript.

These observations are consistent with the results of clinical studies. Decitabine (hypomethylating agent) is effective in 30%–40% of patients with BC-CML [16,113,114,115,116]. The combination of decitabine and the TKI dasatinib in the blast phase induces better therapeutic responses than TKI alone [117]. The response rate in CP-CML is higher, although transient. This effect in the early stages of the disease is consistent with the existence of altered DNA methylation patterns already at diagnosis [77]. Furthermore, the intra-clonal heterogeneity of DNA methylation and the existence of a specific methylation profile of CD34+ cells containing TKI-resistant LSCs suggest that the combination of hypomethylating agents with TKIs in the early stages of the disease might be an interesting strategy to target TKI-resistant LSCs. However, the myelosuppression induced by these drugs and the constraints related to injectable forms remain an ethical obstacle to the implementation of a clinical research protocol in patients with CP-CML because of the benefit/risk balance and the difficulty to formally identify patients with CML that will be resistant or less sensitive to TKIs. New drugs or dosage forms, especially oral ones, which have already proven their efficacy in AML and myelodysplastic syndromes [118,119,120] could be an interesting alternative.

### 5.3. DNA Methylation as a Biomarker of TKI Resistance and Intra-Clonal Heterogeneity

The DNA methylation profile of cancer or leukemia cells can be correlated with prognosis. For instance, in chronic lymphocytic leukemia, three prognostic subgroups, identified on the basis of DNA methylation data, globally correlate with other prognostic factors [121,122,123,124,125]. In CML, several studies have identified DNA methylation changes in the promoter of some genes in the advanced phases of the disease, for instance hypermethylation of the *ABL1* [80], cell cycle-regulating genes [43], CEBPA [81], *PU-1* [79], *CALCA* [82], HOXA4 or HOXA5 [63] gene promoters. Moreover, global DNA methylation studies have found a clear increase in DNA methylation alterations in BC cells. As advanced CML phases are more resistant to TKIs, some DNA methylation abnormalities could be correlated with therapeutic resistance. Moreover, it has been reported that some DNA methylation changes correlate with imatinib resistance, such as hypermethylation of *HOXA4* [63], *BIM* [83], OSCP1 or NPM2 [38]. These observations suggest that some DNA methylation marks may be related to TKI susceptibility and/or resistance. Subclonal evolution may emerge under treatment, but only four genes have been related to treatment resistance thus far. More studies are needed to identify other DNA methylation marks.

Nevertheless, this intra-clonal heterogeneity might be a less important factor of resistance in some patients. Indeed, 10%–15% of patients can be considered cured in the long term, i.e., with undetectable disease after stopping treatment. This implies that in patients in long-term treatment-free remission, all cells were at some point sensitive to TKIs. It also suggests that intra-clonal heterogeneity and treatment response show inter-individual variability. However, this only concerns a minority of patients. All these observations suggest that DNA methylation alterations and intra-clonal heterogeneity could represent biomarkers of TKI resistance in the early phase of CML. More studies are required to identify the methylation marks associated with TKI-resistant cells in vivo.

## 6. Conclusions

CML is a particularly interesting model to discover new mechanisms of resistance to targeted therapies and biomarkers of predisposition to the development of acute leukemia, besides CHIP and gene mutations. Epigenetic regulatory perturbations are emerging as novel candidate mechanisms, including DNA methylation.

Studies in CML cells show that the presence of the t(9;22) translocation is accompanied by disturbances in the DNA methylation pattern that are clearly amplified during disease progression. More recently, intra-clonal differences in DNA methylation have been detected already at diagnosis and several observations indicate that some DNA methylation alterations are probably BCR-ABL-independent. These findings open complementary research perspectives to (1) better understand the pathophysiological mechanisms of the establishment of these abnormalities throughout the disease course, (2) identify the methylation abnormalities correlated with resistance to TKIs or disease progression and (3) evaluate the interest of epigenetic drugs in CP-CML.

These different research avenues will ultimately allow for optimizing the management of each patient to increase the number of patients who can be cured and to find therapeutic solutions for CML progression.

## Figures and Tables

**Table 2:** DNA methylation alterations and intra-clonal heterogeneity.

DNA Methylation Alterations	Observations	Main Contribution
Genetic driver: BCR-ABL1	BCR-ABL1 induces DNA hypomethylation through DNMT1 overexpression in mice [95] BCR-ABL1 induces an aberrant DNA methylation profile that is reversed upon BCR-ABL1 repression in mice [94] Disappearance of DNA methylation abnormalities at remission after TKI treatment in patients with CP-CML [92]	Impact of BCR-ABL on DNA methylation of the CML clone
Inter-individual variability	DNA methylation variation profile in different samples at diagnosis [34,44,77]	Inter-patient variability of DNA methylation suggesting BCR-ABL- independent mechanisms
Influence of cell origin at BC transformation	Myeloid and lymphoid blast phase cells have similar methylation profiles [34]	DNA methylation modification is more dependent on LSCs than cell lineage commitment
Mutations in DNA methylation regulators	5% in CP-CML [32,62,76,84,85,86,87] 16% of AP/BC-CML [51]	Mutations in DNA methylation regulators are found in a minority of patients and in a fraction of the CML clone
Differentiated vs immature CML cells	DNA methylation alterations specific of immature and mature cells [34,77]	Intra-clonal heterogeneity of DNA methylation Possible influence of stem cell status on DNA methylation
Polycomb complex (EZH2)	Implication in transformation to the blast phase through DNA hypermethylation [34]	EZH2 is involved in CML aggressiveness through DNA methylation

## Data Availability

The HM450K DNA methylation data used to generate the DNA methylation clock are available at NCBI GeneExpression Omnibus (GEO; http://www.ncbi.nlm.nih.gov/geo/) under accession number GSE106600 since march 2018.
